# Secondary Structural Model of Human MALAT1 Reveals Multiple Structure–Function Relationships

**DOI:** 10.3390/ijms20225610

**Published:** 2019-11-09

**Authors:** Phillip J. McCown, Matthew C. Wang, Luc Jaeger, Jessica A. Brown

**Affiliations:** 1 Department of Chemistry and Biochemistry, University of Notre Dame, Notre Dame, IN 46556, USA; pmccown@nd.edu (P.J.M.); mwang7@nd.edu (M.C.W.); 2 Department of Chemistry and Biochemistry, Biomolecular Science and Engineering Program, University of California at Santa Barbara, Santa Barbara, CA 93106, USA; jaeger@ucsb.edu

**Keywords:** long noncoding RNA, secondary structure, MALAT1, m^6^A, cancer

## Abstract

Human metastasis-associated lung adenocarcinoma transcript 1 (MALAT1) is an abundant nuclear-localized long noncoding RNA (lncRNA) that has significant roles in cancer. While the interacting partners and evolutionary sequence conservation of MALAT1 have been examined, much of the structure of MALAT1 is unknown. Here, we propose a hypothetical secondary structural model for 8425 nucleotides of human MALAT1 using three experimental datasets that probed RNA structures in vitro and in various human cell lines. Our model indicates that approximately half of human MALAT1 is structured, forming 194 helices, 13 pseudoknots, five structured tetraloops, nine structured internal loops, and 13 intramolecular long-range interactions that give rise to several multiway junctions. Evolutionary conservation and covariation analyses support 153 of 194 helices in 51 mammalian MALAT1 homologs and 42 of 194 helices in 53 vertebrate MALAT1 homologs, thereby identifying an evolutionarily conserved core that likely has important functional roles in mammals and vertebrates. Data mining revealed that RNA modifications, somatic cancer-associated mutations, and single-nucleotide polymorphisms may induce structural rearrangements that sequester or expose binding sites for several cancer-associated microRNAs. Our findings reveal new mechanistic leads into the roles of MALAT1 by identifying several intriguing structure–function relationships in which the dynamic structure of MALAT1 underlies its biological functions.

## 1. Introduction

Over 15,000 long noncoding RNAs (lncRNAs) have been discovered in humans, yet little is known about their structure and function [1,2]. Metastasis-associated lung adenocarcinoma transcript 1 (MALAT1), also known as nuclear-enriched abundant transcript 2 (NEAT2), is an 8708-nucleotide (nt) lncRNA that is conserved in vertebrates and localizes to nuclear speckles [3,4]. Aberrant MALAT1 expression is implicated in cancer progression and in metastasis [5,6,7]. How MALAT1 coordinates these various processes is gradually being elucidated from the interacting partners of MALAT1, such as proteins, U1 small nuclear RNA (snRNA), and microRNAs (miRNAs or miRs) [7,8,9,10,11,12,13,14,15]. Roles for MALAT1 in alternative splicing are mediated via interactions with serine/arginine (SR) splicing factors, heterogeneous nuclear ribonucleoprotein (hnRNP) C and hnRNPG, and the U1 snRNA [8,11,12,13]. Furthermore, MALAT1 acts as a competitive endogenous RNA, whereby miRNAs are sponged by MALAT1 rather than their mRNA targets [7,16]. For example, increased MALAT1 expression leads to sponging of miR-217 in pancreatic cancer cells, increasing oncogenic *KRAS* expression [16]. Collectively, these interactions with proteins, U1 snRNA, miRNAs, and other factors are likely modulated in part by the MALAT1 structure. While the structure of MALAT1 is largely unknown, four local structures have been determined to date. These structures include hairpins at nts 2509–2537 and 2556–2586, which are binding sites for hnRNPG and C, respectively [11,12]. At the 3′ end of MALAT1, a triple helix protects mature MALAT1 from a rapid nuclear RNA decay pathway, while a tRNA-like structure, known as MALAT1-associated small cytoplasmic RNA (mascRNA), facilitates 3′-end processing by RNase P [17,18]. Both the triple helix and mascRNA are highly conserved structures that enabled the discovery of unannotated MALAT1 homologs and MALAT1-like lncRNAs [4,19,20]. 

Various in vitro and in vivo high-throughput RNA structural mapping techniques have been used to model the secondary structures of human lncRNAs [2]. One method is parallel analysis of RNA structure (PARS) whereby RNA is extracted from cells and folded in vitro [21]. RNA is then treated with either RNase S1, which cleaves unstructured or single-stranded RNA (ssRNA), or RNase V1, which cleaves structured or double-stranded RNA (dsRNA) before the digested RNA is subjected to RNA-sequencing (RNA-seq) to identify unstructured and structured regions in the transcriptome [21]. Another method, dimethyl sulfate-sequencing (DMS-seq), uses DMS to chemically modify unpaired adenosine and cytidine residues in vivo [22]. Harvested RNA then undergoes RNA-seq. DMS-reacted bases cause reverse transcriptase to stop, which indicates the presence of unprotected ssRNA [22]. DMS-seq has been used to model the structures of HOTAIR, *Xist*, and NEAT1 lncRNAs [23,24,25]. Methods have also been developed to identify RNA–RNA interacting partners. Psoralen analysis of RNA interactions and structures (PARIS) identifies both intra- and intermolecular RNA-RNA interactions in vivo [26]. A cell-permeable psoralen derivative, 4-aminomethyl-triloxsalen, intercalates into and photocrosslinks to dsRNA upon exposing living cells to UV light [26,27]. The crosslinked RNAs are protected from RNase S1 digestion and subjected to RNA-seq. PARIS has been useful at mapping long-range dsRNA interactions in vivo [26]. These methods and similar ones provide a foundation for modeling lncRNA secondary structures [21,22,23,24,25,26,27,28,29,30,31].

Here, we constructed a secondary structural model of human MALAT1 using PARS, DMS-seq, and PARIS datasets along with a minimum free energy (MFE) computational analysis [21,22,26,27,32]. A phylogenetic sequence analysis of 53 MALAT1 homologs from human through non-mammalian vertebrates revealed that 42 helices out of 194 are found in vertebrates. Furthermore, we mapped known protein-binding sites, intermolecular RNA interaction sites for miRNAs and other RNAs, RNA modifications, somatic cancer-associated mutations, and single-nucleotide polymorphisms (SNPs) to relate structure to prior functional observations of MALAT1. Most notably, this analysis revealed a long-range intramolecular RNA–RNA interaction whose formation likely depends upon the methylation of A5044 and would sequester binding sites for two cancer-associated miRNAs, miR-101 and miR-217. With a working secondary structural model of human MALAT1, this study provides a platform to begin experimentally testing structure–function relationships potentially implicated in cancer and/or metastasis.

## 2. Results

### 2.1. A Secondary Structural Model of Human MALAT1 Was Built Using Multiple RNA Structural Probing Datasets and MFE Calculations

**Figure 1:**
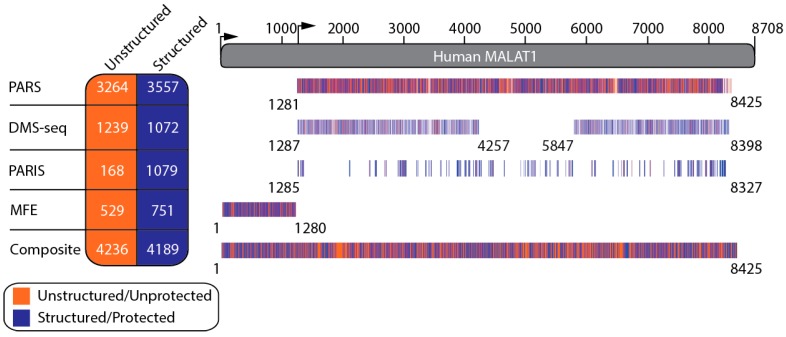
Overview of structural assignments for each nucleotide in human MALAT1. PARS data [21], DMS-seq data [22], PARIS data [26,27], and minimum free energy (MFE) calculations [29,32] were collectively used to construct a composite structural landscape for each nucleotide in MALAT1 (nts 1–8425). The orange lines represent individual nucleotide positions that were designated as unstructured based on PARS datasets and MFE calculations and unprotected adenosine or cytidine residues based on the DMS-seq dataset. The blue bars represent individual nucleotide positions that were designated as structured based on PARS datasets and MFE calculations and as protected adenosine or cytidine residues based on the DMS-seq dataset. The number of nucleotides that are unstructured/unprotected (orange column) or structured/protected (blue column) for PARS, DMS-seq, PARIS, MFE, and the composite model are listed in the table to the left. PARIS data that overlap with unstructured or structured regions of the MALAT1 transcript are presented as either orange or blue, respectively. The short arrow represents the transcription start site of the full-length *malat1* gene (nts 1–8708), while the tall arrow represents the transcription start site of the major variant of the *malat1* gene (nts 1284–8708).

**Figure 2:**
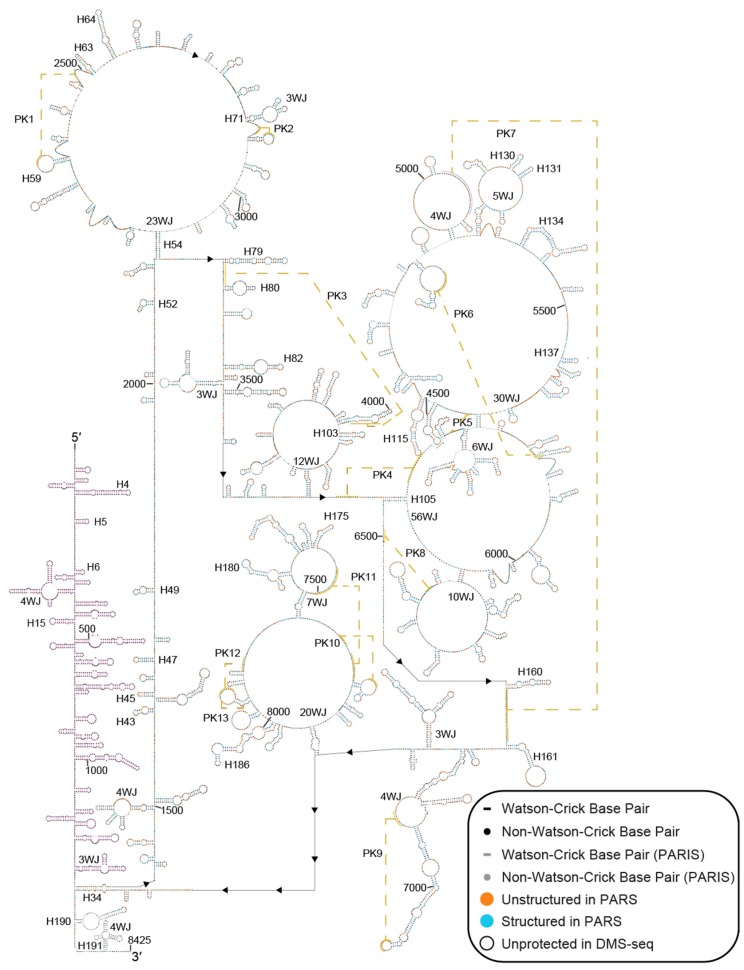
The secondary structural model of human MALAT1. The secondary structures for the composite model are shown with PARS, DMS-seq, and PARIS data annotated as follows: Watson–Crick base pairing (line) and non-Watson–Crick base pairing (dots) are black unless detected by PARIS (gray lines and dots); nucleotide positions with orange and blue circles represent nucleotides that were unstructured and structured, respectively, in PARS and the black outline denotes adenosine and cytidine residues considered unprotected in DMS-seq. The structure of nts 1–1280 (purple) was determined only using MFE calculations from RNAfold [32]. Secondary structures are labeled as follows: H for helix, PK for pseudoknot, and multiway junction as WJ, which is preceded by the number of junctions (e.g., 3WJ for a three-way junction). Each secondary structure is numbered in order of appearance from 5′ to 3′. For clarity, only helices specifically mentioned in the text are labeled; see Table S1 for a complete list of named structures. The solid lines with an arrowhead in the middle of the line denote 0 nt distance. The yellow dashed lines indicate RNA–RNA interactions in helices or pseudoknots. SAVoR and VARNA were used to visualize the model [33,34].

To create a secondary structural model of human MALAT1, we first examined the PARS and DMS-seq datasets from noncancerous human lymphoblastoid and fibroblasts cells, respectively (see Methods) [21,22]. MALAT1 reads were extracted from these datasets and used to model the secondary structure of four known structures in MALAT1: the hnRNPC- (nts 2556–2586) and hnRNPG-binding hairpins (nts 2509–2537), the triple helix-containing hairpin (nts 8263–8355) and the tRNA-like structure of mascRNA (nts 8356–8412) (Figure S1) [11,12,17,18]. For nucleotides that have data in PARS and DMS-seq datasets, the PARS datasets correctly predicted 70.7% (87 of 123 nts) of the nucleotides known to be ssRNA or dsRNA, while the DMS-seq dataset correctly predicted 72.6% (45 of 62 adenosine or cytidine nucleotides) known to be ssRNA or dsRNA (Figure S1). From this test, two key findings emerged: (i) these structures demonstrated that PARS and DMS-seq datasets can be used to build a reliable secondary structural model of MALAT1 and (ii) this analysis established a scoring cutoff to assign nucleotides in MALAT1 as structured/protected or unstructured/unprotected (see Methods; Figure 1 and Table S1). The resulting MALAT1 structural model was refined with the addition of 95 RNA duplexes determined by PARIS in HEK293T cells [26,27]. Together, these PARS, DMS-seq, and PARIS datasets provided structural information on 79% of full-length human MALAT1 (6879 of 8708 nts) (Figure 1 and Figure 2 and Figure S2). Because the PARS and DMS-seq datasets provide structural information on nts 1281–8425 of MALAT1, we used RNAfold, a program in the ViennaRNA package that calculates MFE structures, to predict the local secondary structure of nts 1–1280 (Figure 1) [29,32]. Our composite secondary structural model of MALAT1 (nts 1–8425) is presented in Figure 2. When constructing the secondary structure of MALAT1, we encountered instances where MALAT1 appeared to be structured based on structure probing data but could not identify a discernible base-pairing partner. Therefore, we listed these regions as being unstructured in the final model (see Methods and Figure 2). These regions may represent portions of MALAT1 that are bound by protein(s), may participate in unidentified base-pairing interactions (i.e., intra- and intermolecular RNA-RNA interactions), or yielded ambiguous results inherent to the PARS, DMS-seq and/or PARIS methods.

Overall, our model of MALAT1 predicts that ~50% of nucleotides are engaged in secondary structures (Figure 1 and Figure 2). Within our model, the single-stranded and double-stranded characteristics of the PARS, DMS-seq, and PARIS data agree with 60%, 56%, and 100%, respectively, of the structural assignments in the composite model (Table S1). Inconsistencies between the PARS and DMS-seq data are not surprising considering that PARS examines RNA structure folded in vitro, whereas DMS-seq examines RNA structure inside living cells [21,22]. Furthermore, DMS failed to modify protein-bound ssRNAs in vivo and was subsequently interpreted as structured; therefore, this limitation may account for some of the inconsistencies with the DMS-seq data (Table S1) [22]. 

**Figure 3:**
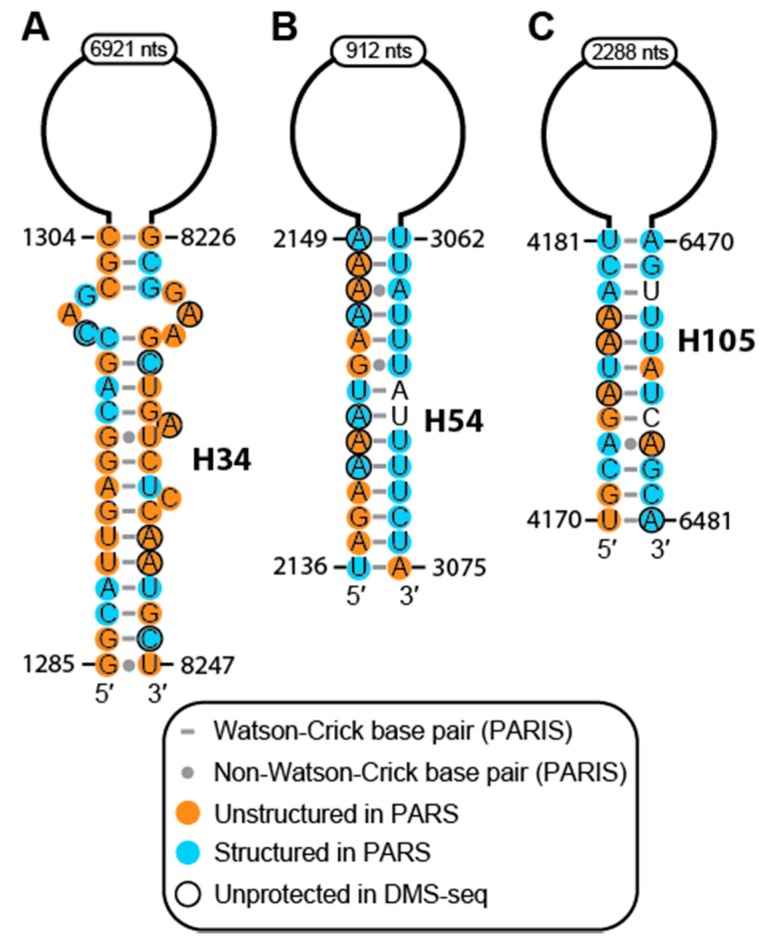
The secondary structural models for three long-range helices. The secondary structures are shown for PARIS-identified helices: (**A**) H34, (**B**) H54, and (**C**) H105 [21,22,26,27]. All PARS, DMS-seq, and PARIS data are annotated as described in Figure 2.

Our proposed model consists of 194 helices, 13 pseudoknots (PK), four three-way junctions, five four-way junctions, one five-way junction, and eight multiway junctions (Figure 2 and Table S1). We also observed seven consensus UAA-GAN internal loops, one consensus kink-turn, three consensus U-turns, one GNRA tetraloop, one ANYA tetraloop, and one loop E motif (Figure 2 and Table S1). PARIS data were particularly important in assigning MALAT1 structural features to dsRNA regions spanning distances >100 nts (Figure 3) [26,27]. For example, H34 forms a 17-base-paired (bp) duplex with a 3-nt internal loop from two regions of MALAT1 separated by 6921 nts in the MALAT1 transcript (Figure 2 and Figure 3A). While H34 appears in PARIS datasets and is further supported by the DMS-seq dataset, the large number of single-stranded nucleotides present in the PARS dataset may suggest that H34 does not form under the in vitro refolding conditions used in preparing RNA for PARS [21,22,26,27]. The hnRNPG- (H63) and hnRNPC-binding hairpins (H64) are predicted to reside within a large, multi-stem domain that is contained within the H54 loop (Figure 2 and Figure 3B) [11,12]. In addition, H105 forms a 12-bp duplex from two regions of MALAT1 separated by 2288 nts in the MALAT1 transcript, which encompasses 54 helices and five pseudoknots (Figure 2 and Figure 3C). Thus, the combined structural analyses of the PARS, DMS-seq and PARIS datasets revealed a model containing both the local and global secondary structures of MALAT1. 

### 2.2. Conservation and Covariation Analyses Identified Evolutionarily Conserved Features in MALAT1 Homologs

Next, we examined the structural conservation of MALAT1 to support our secondary structural model of human MALAT1. First, a consensus structure was built from the MALAT1 sequences of human, bonobo, bushbaby, mouse, and cat (see Methods and File S2). This alignment was then used by Infernal to search for homologous structures in 53 annotated MALAT1 sequences: 17 from primate species, 51 from mammalian species, and 53 from vertebrate species. The overall nucleotide identity is 28%, 25%, and 25% for primates, mammals, and vertebrates, respectively. Our analysis of secondary structures revealed that 153 out of 194 helices were conserved with at least 90% of the helix intact in at least 43 mammalian MALAT1 homologs and with at least 75% nucleotide conservation (see Methods and Figure S2 and Files S3–S14). Expanding our analysis to vertebrates, we identified an evolutionary core of 42 helices in the 53 MALAT1 homologs examined, spanning nts ~4500–6700 and ~8000–8425 (Figures S2 and S3, Files S3–S14 and Table S1). All the PKs in our model are conserved in mammals while only PK6-8 are conserved in non-mammalian vertebrates (Table S1). An example of a highly conserved helix is H131 (nts 5203–5220 in human); it is found in 50 MALAT1 homologs and shows covariation or co-mutation in all seven base pairs (Figure S4). Another region, spanning H160–H161 (nts 6550–6800), is present in all 53 MALAT1 homologs (Figure S3). These evolutionarily conserved structures further corroborate our model and could aid in finding new MALAT1 homologs as well as discerning between MALAT1 and MALAT1-like homologs [20].

The statistical significance of secondary structural models of other lncRNAs has been examined using R-scape; therefore, we conducted this analysis on our MALAT1 secondary structure using the newly revised R-scape parameters reported by Tavares et al. [23,24,25,28,35,36]. R-scape identified 40 statistically significant covarying base pairs out of 1932 helical base pairs throughout our entire MALAT1 secondary structure, including the evolutionary landmarks of the triple helix and mascRNA structures (Table S1). However, this lack of statistical significance of covarying nucleotides in MALAT1 is similar to that reported for the HOTAIR, *Xist*, SRA, and NEAT1 lncRNAs [23,24,25,28,35,36]. Regions of MALAT1 have been investigated in several evolutionary studies for the presence of conserved structures, which have been found consistently despite the use of different computational methods [4,19,20,37]. Therefore, the poor statistical significance calculated by R-scape may be indicative of MALAT1 not having enough evolutionary time to optimize structural features or sample structural space, as was hypothesized for the *neat1* gene, which is adjacent to the *malat1* gene, and for other lncRNAs [24,25,35,36].

### 2.3. The MALAT1 Structural Model is Consistent with Known Protein-Binding Motifs

**Figure 4:**
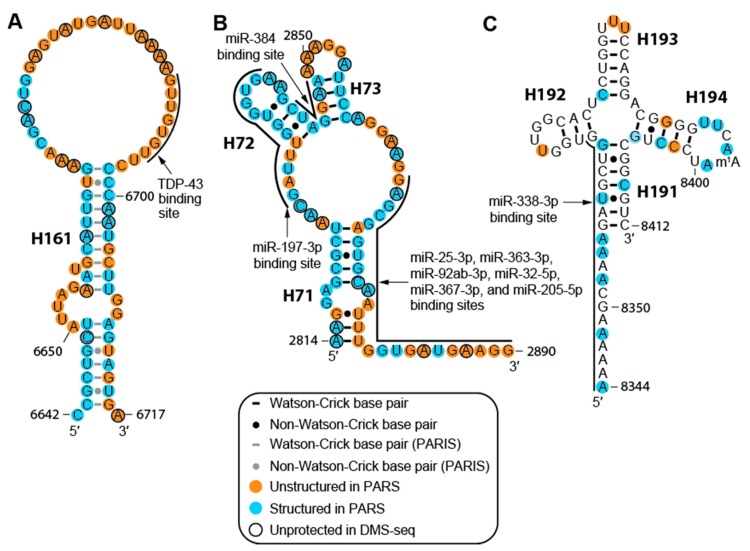
Select protein- and miRNA-binding sites mapped onto the MALAT1 structural model. (**A**) The TDP-43 binding site at the predicted loop of H161 consists of single-stranded UG/GU repeats [10]. (**B**) H71-H73 have eight miRNA-binding sites. Of these, miR-25-3p, miR-205-5p, and miR-363-3p have been experimentally validated, while the other miRNA binding sites are predicted based on starBase, a repository of putative and experimentally validated interacting partners of RNAs [15,44,45,46]. (**C**) Select triple helix-forming nts (8345–8355) and the 5′ stem of H191 contain a validated binding site for miR-338-3p [47]. All PARS, DMS-seq, and PARIS data are annotated as in Figure 2.

MALAT1 has more than 410 putative protein-binding partners in HeLa cells based on capture hybridization analysis of RNA targets (CHART) and over 120 putative binding partners in HepG2 liver cancer cells based on quantitative proteomics approaches and in PC3 prostate cancer cells based on hybridization purification of RNA-protein complexes followed by mass spectrometry (HyPR-MS) [38,39,40]. Of these binding partners, human antigen R (HuR), hnRNPC, hnRNPG, methyltransferase-like proteins 3 and 14 (METTL3/14), METTL16, and TAR DNA-binding protein 43 (TDP-43) have binding sites for specific sequences and structures characterized at single-nt resolution [9,10,11,12,41,42]. Importantly, our structural model is consistent with the known binding motifs or sites of these proteins (Figure 2 and Table S2). A TDP-43 binding site containing the characteristic single-stranded GU/UG repeats resides in the loop of H161 (Figure 4A) [10]. hnRNPC binds to H64 [11] and hnRNPG binds to H63 [12] (Figure S1 and Table S2) while HuR binds to H79, H82, and H115 (Table S2). METTL3/14 binds to nine different RRACH sequence motifs (where R = purines and H = any nucleotide but guanosine) in MALAT1 [43]. Furthermore, METTL16 binds to the 3′-terminal triple helix [41]. Most of these protein-binding sites are conserved in MALAT1. The sequences found in the binding sites for hnRNPC, hnRNPG, HuR, METTL3/14, and TDP-43 appear in almost every primate and 36 mammalian MALAT1 homologs examined (Table S2). The METTL16-binding site, which is the highly conserved triple helix, is present in every MALAT1 homolog [4,20]. Because the binding sites of these seven proteins are found in MALAT1 homologs, their presence not only supports regions of our MALAT1 structural model but also demonstrates that these proteins may facilitate important functions of MALAT1 in multiple species. 

While we emphasized several validated MALAT1–protein interactions, there are hundreds more predicted protein-binding sites that may elucidate structural features in MALAT1 [38,40]. For example, consensus sequences for binding sites of Lin28A, serine/arginine-rich splicing factor (SRSF)1, and SRSF10 were found between H49 and H52 (nts 1876-2052) using the Tomtom module in MemeSuite (data not shown) [48]. As this region between H49 and H52 is largely devoid of structure, it is possible that this region may be primed for binding to Lin28A, SRSF1, and SRSF10, although these binding sites have not been verified to date. We expect that further characterization of protein-binding sites on MALAT1 will shed more light on this working secondary structural model.

### 2.4. Structure of MALAT1 May Regulate Binding-site Accessibility for Diverse Classes of RNAs

In addition to protein-binding partners, MALAT1 forms intermolecular RNA–RNA interactions with U1 snRNA, rRNA, and ribosomal protein S6 (RPS6) mRNA (Table S2) [13,31]. The U1, rRNA, and RPS6 RNA sites that uniquely interact with MALAT1 are present in nearly all primates and most of the mammalian species examined herein. Three out of seven U1 snRNA-binding sites were found in vertebrate MALAT1 homologs while the other four sites are exclusive to mammals (Table S2). Interestingly, the U1 snRNA-binding site at nts 3152–3186, which is unpaired in our model, overlaps with an HuR-binding site between H79 and H80 (Figure 2 and Table S2), which suggests that U1 snRNA and HuR could compete for this binding site [9,13].

Furthermore, human MALAT1 is predicted to bind at least 114 miRNAs in at least 152 different sites (Table S2). All miRNA-binding sites are conserved in mammals, although a few (e.g., miR-338-3p, miR-217-5p, miR-101-3p, and miR-383-5p) are present in non-mammalian vertebrates (Table S2). Of the 114 miRNAs, 43 have been experimentally validated using a combination of cell-based luciferase or GFP reporter assays, knockout, knockdown, or mutation-based assays (Table S2) [15]. While 125 of 152 miRNA-binding sites occur in regions of MALAT1 that we predict to be structured in normal cells (Table S2), both PARS and DMS-seq analyses have indicated that their respective data do not adequately account for dynamic structural features [21,22]. What may be structured in our analyses may be unstructured in other cellular environments, namely different cell and cancer types. For example, in H71-H73 and PK2, there are eight miRNA-binding sites, including miR-25-3p, miR-205-5p, and miR-363-3p as confirmed interacting partners of MALAT1 (Figure 4B and Table S2) [15,44,45,49]. Moreover, miR-25-3p, miR-32-5p, miR-92-3p, miR-363-3p, and miR-367-3p belong to the miR-25 family, which is conserved through vertebrates, including several that do not have MALAT1 homologs presently known [50,51]. The number of miRNAs with binding sites in H71-73 suggests that this region may mediate unique roles depending on the cellular context [15,46]. The miR-25 miRNA family has the same targets; however, overexpression of these miRNAs can be either oncogenic or tumor-suppressive, depending on the cancer type [46,49]. Similarly, miR-205-5p overexpression is linked to increased proliferation and invasion in ovarian cancer cells, whereas miR-205-5p overexpression was linked to tumor-suppressive effects by decreasing MALAT1 levels in renal cell carcinoma and osteosarcoma [44,52,53]. Finally, a validated miR-338-3p binding site resides in the triple helix and mascRNA region (H190-H191) (Figure 4C); therefore, this miRNA may interfere with the formation of the triple helix and mascRNA, which would subsequently affect the overall stability and 3′-end processing of MALAT1 [17,18,47]. Thus, the structure of MALAT1 may represent an important paradigm in regulating miRNA-binding site accessibility akin to a rheostat, which can affect the cellular function of healthy cells and various disease states. 

### 2.5. RNA Modifications Alter the Structure and RNA-interacting Partners of MALAT1

**Figure 5:**
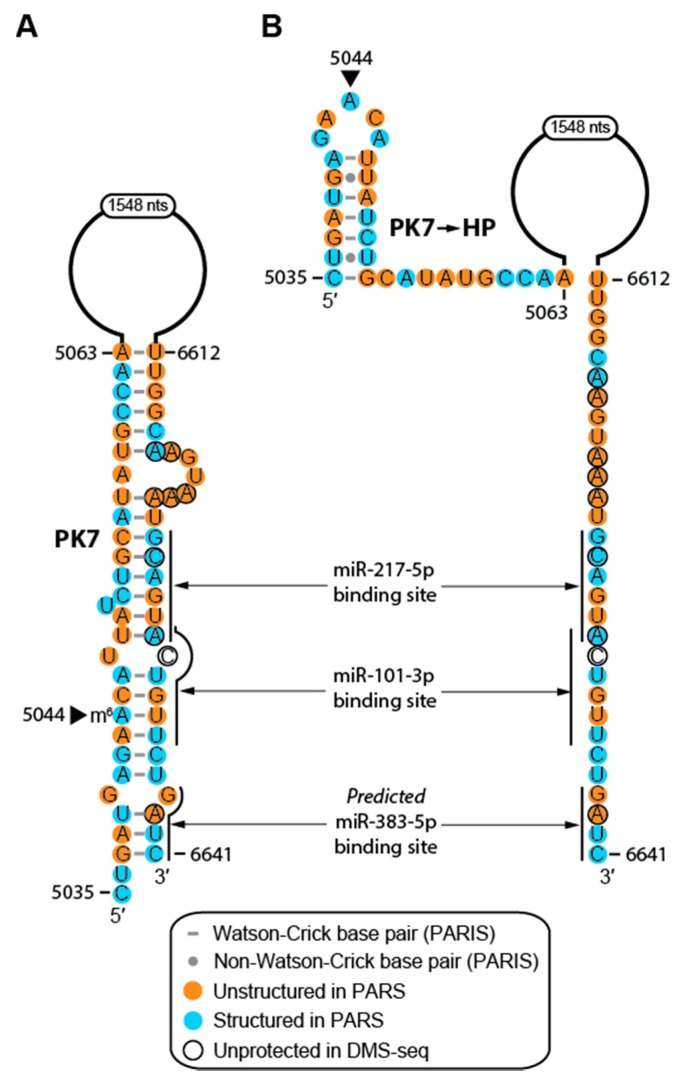
m^6^A status of A5044 differentially regulates the structure of PK7 in human cells. (**A**) PK7 is a helix that bridges 1548 nts in MALAT1 and was detected by PARIS in HEK293T cells. m^6^A5044 (marked by a caret, ►) was observed by prior (m^6^A/MeRIP-seq) experiments in HEK293T cells [43]. Binding sites for miR-217-5p, miR-101-3p, and the predicted binding site for miR-383-5p were determined from prior experiments [15,16,61,62]. (**B**) PK7→HP is the predicted structure that forms based on PARIS experiments in HeLa cells [26,27]. The m^6^A5044 mark has not been detected in HeLa cells [43]. All PARS, DMS-seq, and PARIS data are annotated as described in Figure 2.

RNA modifications dynamically regulate RNA structures, and multiple RNA modifications have been identified in human MALAT1 to date [11,12,54,55,56,57,58,59]. Therefore, we mapped the following modifications determined at single-nt resolution onto the MALAT1 secondary structure: 12 *N*^6^-methyladenosines (m^6^A), one *N*^1^-methyladenosine (m^1^A), three pseudouridines (Ψ), seven 5-methylcytidines (m^5^C), one *N*^7^-methylguanosine (m^7^G), and three 2′-*O*-methylated nucleotides (Nm) (Table S2) [54,55,56,57,58,59]. At least three m^6^A modifications appear to affect the secondary structure of MALAT1 and function as structural switches. Prior studies have shown that m^6^A marks in H63 and H64 (m^6^A2515 and m^6^A2577, respectively) of MALAT1 create binding sites for hnRNPC and hnRNPG by destabilizing hairpin structures (Figure S1) [11,12]. Interestingly, we found another m^6^A mark, m^6^A5044 in PK7, which may modulate the structure of MALAT1 in different cellular contexts. In normal human cells (e.g., HEK293T), m^6^A5044 may facilitate the formation of PK7, which is a pseudoknot between nts 5037–5063 and nts 6612–6641 that was detected by a relatively strong signal of eight reads in one of three PARIS datasets collected from HEK293T cells (Figure 2 and Figure 5) [26,27,42]. In HeLa cells, A5044 is not methylated based on m^6^A-methyl-RNA-immunoprecipitation sequencing (m^6^A/MeRIP-seq) results [43] and the PARIS-identified PK7 that is found in HEK293T cells is absent [26,27]. Instead, both PARIS datasets from HeLa cells detected a local hairpin structure, which we denote as PK7→HP (Figure 5B). Because m^6^A has been known to both stabilize and destabilize RNA structures, m^6^A5044 may stabilize PK7, a structure that appears to be transient because it is detected in only one of three PARIS datasets [11,12,26,27,60]. Unfortunately, long-range RNA interaction studies have not been performed on additional cell lines to further support the presence of PK7 in normal cell types and PK7→HP in cancer. However, the m^6^A5044 mark has not been detected in several human cancer cell lines, notably acute myelogenous leukemia (AML), hepatocellular carcinoma (HepG2), and non-small cell lung cancer (H1299) cells (Table 1) [59]. The variable presence of the m^6^A5044 mark is in contrast to m^6^A2515, m^6^A2577, and m^6^A2611, which are essentially ubiquitous modifications in MALAT1 regardless of cell type (Table 1) [59].

Disruption of PK7 in cancer cells, whether it is caused by m^6^A5044, protein-binding partners, or RNA-binding partners, would expose binding sites for miR-101, miR-217, and miR-383, allowing MALAT1 to sponge these miRNAs [7,16,61]. Interestingly, the 5′ region of PK7 (nts 5035–5063) is at least 80% conserved in 49 out of 53 MALAT1 homologs examined herein, and the 3′ region of PK7 (nts 6612–6641) is at least 90% conserved in 52 out of 53 MALAT1 homologs examined herein (Files S8 and S10 and Figure S3). This high level of conservation suggests that this region mediates a significant structure–function relationship. Thus, we propose an m^6^A switch that may explain one mechanism by which MALAT1 plays a role in cancer and m^6^A5044 as a potential biomarker for cancer.

### 2.6. Cancer-associated Mutations and SNPs May Affect the Structure of MALAT1 

**Figure 6:**
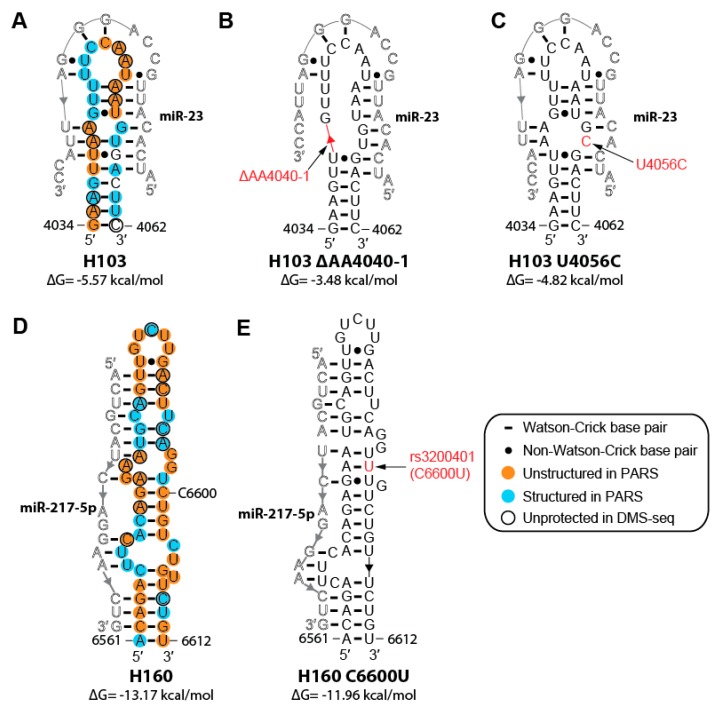
Predicted structural changes of helices and miRNA-binding sites by cancer-associated mutations and by SNP rs3200401. Schematics of the secondary structure of human H103 interacting with miR-23 [65,66] for (**A**) WT MALAT1, (**B**) the A4040/A4041 double deletion (red line) in breast invasive carcinoma (BRCA) [63], and (**C**) the U4056C substitution (red) in uterine corpus endometrial carcinoma (UCEC) [63]. Schematics of the secondary structure of human H160 interacting with miR-217-5p [16] for (**D**) WT MALAT1 and (**E**) the C6600U (red) SNP. All PARS and DMS-seq data are annotated as described in Figure 2. Predicted secondary structures and ΔG values of H103 and H160 mutants were determined using RNAfold [32].

A total of 655 mutations in MALAT1 have been reported for 26 types of cancer [63]. Here, we highlight two mutations that may affect a binding site for the tumor suppressor miR-23abc-3p that is conserved in all mammalian MALAT1 homologs we examined, except for the tarsier homolog (Figure 6A–C, Table S2 and File S6) [64]. One mutation, ΔAA4040-4041, was identified in the breast invasive carcinoma (BRCA) dataset in the Genomic Data Commons while the second mutation, U4056C, was identified in the uterine corpus endometrial carcinoma (UCEC) dataset in the Genomic Data Commons [63]. Both mutations weaken the stability of H103 based on free-energy calculations by RNAfold (Figure 6A–C) [32]. As MALAT1 acts as a competitive endogenous RNA for miR-23 in gastric and pancreatic cancer, destabilization of H103 would increase miRNA-binding site accessibility, making MALAT1 a better miRNA sponge for miR-23 (Figure 6B) [64,65,66]. H103 U4056C would disrupt MALAT1–miR-23 binding in the seed region. While it is unclear how this U4056C mutation in MALAT1 would contribute to the progression of cancer, elevated levels of miR-23 have been shown to increase epithelial-mesenchymal transition (EMT) in cancer cells [67]. Thus, the loss of a miR-23-binding site on MALAT1 could enable miR-23 binding elsewhere in the cell.

Of the 17 annotated SNPs in MALAT1, SNP rs3200401 (C6600U) resides in H160, which contains a binding site for the tumor suppressor miR-217-5p (Figure 6D,E) [16,68]. RNAfold predicts that this SNP may decrease the stability of the H160 hairpin by increasing the number of G•U wobbles from one to two (Figure 6E) [32]. However, this SNP reduces the number of unpaired nucleotides in internal loops, making the miR-217-5p-binding site less accessible. Collectively, these three examples illustrate how only one- and two-nucleotide changes could potentially alter the structure–function relationships of MALAT1, leading to different effects in different disease states.

## 3. Discussion

In this study, we present a working secondary structural model of nts 1–8425 of human MALAT1 using data obtained from PARS, DMS-seq, and PARIS datasets coupled with MFE computations using ViennaRNA [21,22,26,27,32]. Our model of MALAT1 includes 194 helices, 13 pseudoknots, and several regions of multiply nested helical structures resulting from long-range interactions (Figure 2). Our conservation and covariation analyses provided additional support for our model, revealing 42 evolutionarily conserved helices (Figures S2 and S3 and Table S1). Several structured areas within our model (H6-15, H43-48, H59-64, H101-106, H134-142, PK11-H175, and H189-194) were also present in a previous study that identified structured domains, but not specific structures, of MALAT1 (Table S1) [69]. Moreover, several isolated helices predicted in a prior MFE modeling study appear in our model of MALAT1, including helices H5, H46-47, H130-131, H137, and H191-194 as well as portions of H4 (nts 98–128), H45 (nts 1724–1742), and H180 (nts 7714–7749) [19]. Importantly, our model of MALAT1 predicted approximately 180 additional helices and further defined long-range interactions that were not reported in previous MFE-based studies [11]. We defined an evolutionary core for the 53 MALAT1 homologs we examined; this core includes the region spanning H115-H161 and the region spanning H186-H194, which includes the previously established triple helix and mascRNA structures (H190-194) (Figures S2 and S3) [17,18,19,20]. Together, these structures may be useful in identifying other MALAT1 homologs and distinguishing between MALAT1 and MALAT1-like homologs [20]. Lastly, well-characterized MALAT1-protein interactions, most of which are conserved in mammals, provided additional support for our model (Table S2) [9,10,11,12,41,43]. For example, TDP-43, a protein with crucial roles in frontotemporal dementia and amyotrophic lateral sclerosis, binds to a single-stranded UG/GU-rich sequence located in loop H161 of our model (Figure 2 and Figure 4A) [10,70]. Collectively, multiple datasets converging on a cohesive structural model provide a starting point for developing new models of how the structure of MALAT1 relates to its biological functions, especially in cancer.

By establishing a working model of MALAT1, several structure-function relationships emerged and may be further tested experimentally. One interesting structure-function relationship is a ~7000-nt long-range interaction (H34) that brings the 5′ and 3′ ends into closer proximity (Figure 3A), an end-to-end joining that is structurally analogous to what occurs in mRNA [71]. Structures like H34 may be a common feature of other lncRNAs, as similar end-joining helices have been observed in the 3735-nt isoform of NEAT1 [25,71]. Because this end-joining permits protein factors to bind to mRNAs and regulate transcript stability, it is possible that the end-joining observed in MALAT1 serves a similar function [71]. With MALAT1 being implicated in alternative splicing, another intriguing region to investigate is the largely unstructured region between H49 and H52, for Lin28A, SRSF1, and SRSF10 binding-site motifs exist in this region protected from DMS (Figure 2 and Table S1) [48]. Likewise, the function of the U1 snRNA–MALAT1 interactions remains unclear (Table S2). 

Integrating our secondary structural model of MALAT1 with protein- and RNA-binding sites, RNA modifications, cancer-associated mutations, and SNPs unexpectedly revealed new mechanisms by which MALAT1 may function in cancer. Most notably, m^6^A/MeRIP-seq and PARIS datasets allowed us to speculate that A5044 functions as an m^6^A switch, whereby m^6^A5044 stabilizes formation of PK7, which would occlude miRNA-binding sites, and unmethylated A5044 favors formation of PK7→HP, which would allow miR-101 and miR-217 to be sponged by MALAT1 in cancer (Figure 5) [27,42,43]. Sponging of miR-101 and miR-217 is critical for the development or metastasis of esophageal squamous cell carcinoma, pancreatic ductal adenocarcinoma, cervical cancer, lung adenocarcinoma, and colorectal cancer [16,61,62,72]. Thus, finding the putative m^6^A5044 structural switch to modulate miRNA-binding site accessibility in MALAT1 represents an intriguing mechanism to search in other modified RNAs as well as a novel biomarker and anti-cancer drug target. 

Somatic mutations associated with cancer or other diseases may also alter the structure of MALAT1. We highlighted two cancer-associated mutations that could affect the binding of miR-23 to MALAT1, which act as a competitive endogenous RNA for miR-23 and could potentiate cancer (Figure 6A–C) [64]. Additionally, of the 17 annotated SNPs in MALAT1, SNP rs3200401 (C6600ΔU) may affect the binding of miR-217-5p to MALAT1 at H160 (Figure 6D,E). As this SNP has been associated with a longer median survival time in non-small cell lung cancer patients than those without the SNP (Table S2) [68], it is possible that this SNP disrupts binding of miR-217-5p to MALAT1. Such small changes at the sequence level can perturb MALAT1 secondary structure, leading to profound biological consequences. 

An emerging theme from our study is that the structure of MALAT1, which can be perturbed by RNA modifications, mutations in cancer, and SNPs, plays a critical role in mediating MALAT1–miRNA interactions in cancer. We further demonstrated, with the first secondary structural model of the 8425-nt human MALAT1 lncRNA, that biocomputational approaches can be used to deduce working models of lncRNA secondary structures. Our model suggests that various structures and interacting partners may contribute to MALAT1 function beyond being a simple miRNA or protein sponge. Rather, our model suggests that MALAT1, and perhaps many other lncRNAs, may function like a rheostat or an RNA-based regulator of numerous genes based on dynamic interacting partners and dynamic structures of the lncRNA.

## 4. Materials and Methods

### 4.1. Datasets

The secondary structure of human MALAT1 was modeled using the following datasets downloaded from the Gene Expression Omnibus (GEO; Bethesda, MD, USA): data files GSM1226157-GSM1226168 from the PARS experiments (B-lymphocyte cell lines GM12878, GM12891, and GM12892 from GSE50676), data files GSM1297506-GSM1297508 from the DMS-seq experiment (human fibroblasts from GSE45803), and the data files GSM1917753-GSM1917757 from the PARIS experiments (HEK293T and HeLa cells from GSE74353) [21,22,26,27]. SHAPE datasets (GSE74353) from HEK293T cells were not considered because the files did not report sequencing data on MALAT1 [26]. The sources of the protein-binding data are as follows: ELAV1/HuR-binding site data (GSE29780) from HEK293 cells in the GEO [9], hnRNPC and hnRNPG from HeLa cells from Liu et al. [11,12], METTL3/14 from HEK293 cells from Linder et al. [42] and from HEK293T, HepG2, and HeLa cells from Liu et al. [43], METTL16 from HeLa cells from Brown et al. [41], and TDP-43 from A549, YTLMC-9, and L9981 cells from Guo et al. [10]. The U1 snRNA–RNA interaction dataset (GSE55914) from V6.5 cells was obtained from the GEO [13] and the rRNA and mRNA interaction dataset was obtained from HeLa cells from Aw et al. [31]. miRNA-binding sites in human MALAT1 were obtained from starBase v2.0 (Sun Yat-sen University, Guangzhou, China) on 7 May 2018 [15]. To map RNA modifications, we used the crosslink-induced mutation sites dataset for *N*^6^-methyladenosine (m^6^A) marks in HEK293 cells (GSE63753) [42], in HeLa cells [43], and from the m^6^AVar Database (Sun Yat-sen University, Guangzhou, China) [59]. *N*^1^-methyladenosine (m^1^A) marks (GSE97419, GSE70485, and GSE73941) in HEK293T cells were downloaded from the GEO [55]. Pseudouridine (Ψ) and 5-methylcytidine (m^5^C) marks in HeLa cells were obtained from Jacob et al. [54] and Squires et al. [56], respectively. *N*^7^-methyguanosine (m^7^G) marks in HeLa, HepG2, and HEK293T were obtained from Zhang et al. [57]. 2′-*O*-methyl (Nm) marks (GSE90164, GSE1067864, and GSE1067865) in HEK293 and HeLa cells were downloaded from the GEO [58]. Somatic mutations of MALAT1 in cancer patients were obtained from the National Cancer Institute Genomic Data Commons (Bethesda, MD, USA) on 16 May 2018 [63]. SNP data were obtained from the supplemental data from Wang et al. [68]. In the case of datasets that were mapped to the deprecated reference human genomes hg18 or hg19, nucleotides were remapped to hg38, the current reference human genome [73]. Data were processed and visualized using resources made available by the public server at usegalaxy.org (Penn State University, State College, PA, USA) [74] and by the UCSC Genome Browser (University of California-Santa Cruz, Santa Cruz, CA, USA) [73]. 

### 4.2. Secondary Structure Modeling of MALAT1

Previously validated structures of human MALAT1, which are the hairpins recognized by hnRNPC (H64, nts 2556–2586) and hnRNPG (H63, nts 2509–2537) as well as the hairpins in the triple helix (H190, nts 8263–8355) and mascRNA (H191-194, nts 8356–8412), were analyzed using the PARS and DMS-seq data to determine the numerical cutoff appropriate for assigning regions as single- or double-stranded RNA throughout MALAT1 [11,12,17,18,21,22] (Table S1). RNA was designated as single-stranded if the average RNase S1:V1 read counts were greater than 1 and the average DMS-seq read counts were greater than 20. RNA was designated as double stranded if the average RNase S1:V1 read count was less than 1 and the average DMS-seq read counts were less than 20. If there was a discrepancy between PARS and DMS-seq structural assignments, then the RNAfold program in the ViennaRNA package (University of Vienna, Vienna, Austria), coupled with structural data from PARIS, were applied to resolve structural status [29,32]. dsRNA assignments using PARIS must have satisfied one of the following two criteria: (i) PARIS reads (>3) must be present in at least two of three datasets from HEK293T cells or (ii) PARIS reads (>5) must be present in at least one dataset from HEK293T cells [26,27]. If structural status was still unclear, then the nucleotide or region was designated as unstructured. Pseudoknots (PKs) were determined as follows: (i) PARIS detected a nested base-pairing interaction consistent with an H-type PK or (ii) presence of a structured region inside a loop and the presence of a base-paired structured region outside the helical stem conforming to an H-type PK. PARS, DMS-seq, and PARIS data did not cover MALAT1 nts 1–1280; therefore, local secondary structure was predicted using default parameters in RNAfold by scanning in 100-nucleotide increments with 20-nucleotide overlap from a 5′ to 3′ direction, similarly to NEAT1 structural analysis [25,29,32]. For nts 1281–8425, DMS-seq data were absent from nts 4258–5846; therefore, PARS data, PARIS data, and nearest-neighbor rules were used to determine structures [21,22,26,27,29]. Single-nucleotide structural assignments for PARS and DMS-seq data were visualized using SAVoR (University of Pennsylvania, Philadelphia, PA, USA) [33] (http://tesla.pcbi.upenn.edu/savor) from 28 June 2018 to 9 July 2018. Structures were visualized using R2R (Yale University, New Haven, CT, USA) [75] to calculate and to depict evolutionary conservation in structures and using VARNA (Université Paris-Sud, Paris, France) [34] to compile the finished model. All nucleotide numbering for human MALAT1 follows accession NR_002819.2 and ENST00000534336.1.

### 4.3. Comparing Structural Assignments of Nucleotides

Agreement of ssRNA and dsRNA assignments among PARS, DMS-seq, and PARIS datasets was determined as a percentage. For agreement of ssRNA assignments between PARS and DMS-seq datasets, every adenosine and cytidine with DMS-seq coverage between nts 1287–4257 and 5847–8398 was counted (total = 2311). For these adenosine and cytidine residues, instances were counted (i.e., assigned a value of 1) where both PARS and DMS-seq reported these nucleotides as unstructured (Line 17 in “SeqMarkup” tab, Table S1) or structured (Line 18 in “SeqMarkup” tab, Table S1). This count was divided by 2311. The Composite line (Line 16 in “SeqMarkup” tab, Table S1) is the final structural model as presented in Figure 2. Comparing the ssRNA and dsRNA PARS to the Composite was completed by counting the number of times the two lines agreed with each other for both ssRNA and dsRNA annotations (Lines 19–20 in “SeqMarkup” tab, Table S1). Similarly, ssRNA and dsRNA comparison for DMS-seq to the Composite was calculated by counting the times the DMS-seq and Composite lines agreed (Lines 21–22 in “SeqMarkup” tab, Table S1). All PARIS reads that satisfied the aforementioned criteria were used to define the unstructured or structured status of the Composite line. See “Statistics” tab in Table S1 for compilation of final values.

### 4.4. Conservation and Covariation Analyses of MALAT1 Structural Features

Annotated MALAT1 sequences for 55 organisms were downloaded from the UCSC Genome Browser [73] on 18 June 2018 and formatted into a FASTA document (File S1). As an internal negative control, the antisense sequences of zebrafish and coelacanth MALAT1 homologs (ZebrafishReverse and CoelacanthReverse, File S1) [20] and the entire *Methanobrevibacter ruminantium* genome included in the Infernal 1.1.2 software (Harvard University, Cambridge, MA, USA) (Negative Control, File S1) were added to the MALAT1 FASTA document and used in homology searches. Once a secondary structural model of human MALAT1 was finalized, a Stockholm alignment of this structure was manually curated in a text file. The bonobo, bushbaby, mouse, and cat MALAT1 sequences were aligned to the human MALAT1 sequence and secondary structure in the Stockholm file (File S2) using Clustal Omega (European Bioinformatics Institute, Cambridge, United Kingdom) [76]. These sequences were the input for building covariance models using the Infernal 1.1.2 software package [77]. Because input Stockholm files larger than 1000 nucleotides are computationally taxing for Infernal, the MALAT1 sequences were divided into 12 smaller files, ranging from 174 to 1280 nts. These files were used to build covariance models using the default parameters of cmbuild and were calibrated using default parameters of cmcalibrate. Once the human, bonobo, bushbaby, mouse, and cat MALAT1 sequences were calibrated, the resulting covariance models were used to search a collection of 55 annotated MALAT1 sequences for homologous structures using cmsearch in local and global alignment modes with default search parameters. The zebrafish- and coelacanth-annotated MALAT1 homologs were removed from consideration in subsequent analyses, as none of their sequences could be fit to the MALAT1 secondary structural model except for H190-H194. This brought the total number of MALAT1 homologs from 55 to 53. It is possible that the zebrafish- and coelacanth-annotated MALAT1 transcripts may instead be MALAT1-like homologs, as described for Anole lizards [20]. Once cmsearch found related secondary structures that were statistically significant (*p*-value < 0.05), alignments of these sequences and structures were generated using default parameters of cmalign. These alignment outputs (Files S3–S14) were then visualized using R2R; covarying nucleotide assignments were generated using default parameters outlined in the R2R user manual [75]. Conservation of helices was determined by manual inspection, as aided by Jalview 2 (University of Dundee, Dundee, United Kingdom) [78,79]. Helical structures that were found in at least 43 of 53 MALAT1 sequences (81%) were defined as conserved if there was a nucleotide present ≥75% with at least 90% of helix intact. If primate- or mammalian-specific structural features were being examined, then helices needed to be present in at least 12 of 17 primate MALAT1 homologs (>71%) or 42 of 51 mammalian MALAT1 homologs (>82%) to be defined as conserved. In all cases, the presence of a helix was counted as conserved regardless of gaps in the helical sequence, provided the resulting helix was predicted to form by MFE calculations (32). Conservation of nucleotides in terminal loops was not considered. Simultaneously, these alignments from cmalign and R2R were subjected to an alignment file-wide analysis and by sliding window analysis of individual hairpins using an e-value cutoff of 0.05 on the R-scape website (http://eddylab.org/R-scape) (Harvard University, Cambridge, MA, USA) on 13 March 2019 and 23 April 2019 [28,35].

## Figures and Tables

**Table 1:** m^6^A marks in MALAT1 isolated from several human cell lines. All data were obtained from the m^6^AVar database [42,43,59]. “+” represents the presence of an m^6^A mark and “-” represents the absence of an m^6^A mark. Abbreviations for select human cell lines are as follows: CD8T are CD8-positive T cells, GM are GM12878 cells, hESC are human embryonic stem cells, Neuro are neuroprogenitor cells, and PA-HeLa indicates that photoactivatable crosslinks were generated in HeLa cells when preparing RNA for sequencing.

m^6^A	CD8T	GM	HEK 293T	hESC	Neuro	A549	AML	H1299	HepG2	HeLa	PA-HeLa
**1763**	-	-	+	-	-	-	-	-	-	-	-
**2414**	-	-	+	-	-	-	-	-	-	-	-
**2515**	+	-	+	+	+	+	+	+	+	+	-
**2577**	+	+	+	+	+	+	+	+	+	+	-
**2611**	+	+	+	+	+	+	+	+	+	+	+
**2720**	+	-	+	-	-	+	-	-	-	+	+
**3752**	-	-	+	-	-	-	-	-	-	-	-
**4457**	-	-	+	-	-	-	-	-	-	-	+
**5044**	+	-	+	-	+	+	-	-	-	-	-
**6924**	-	-	+	-	-	-	-	-	-	-	-
**8181**	+	-	+	-	-	-	-	-	-	-	-
**8290**	-	-	+	-	-	-	-	-	-	-	-
	**Normal**					**Cancer**					

## Data Availability

The data that support the findings of this study are openly available at the Gene Expression Omnibus at http://www.ncbi.nlm.nih.gov/geo using GSE or GSM numbers as indicated within the article and/or its supplementary materials.

## Supplementary Materials

Supplementary materials can be found at https://www.mdpi.com/1422-0067/20/22/5610/s1.
