# Supplementary material for: Secondary Structural Model of Human MALAT1 Reveals Multiple Structure–Function Relationships
Source: Int J Mol Sci. 2019 Nov 9;20(22):5610. doi: 10.3390/ijms20225610 (PMC6888369; doi:10.3390/ijms20225610)
Supplement: Supplementary file 1 [file ijms-20-05610-s001.zip › ijms-637661-supplementary_figures.docx]

**Supplementary Figures**


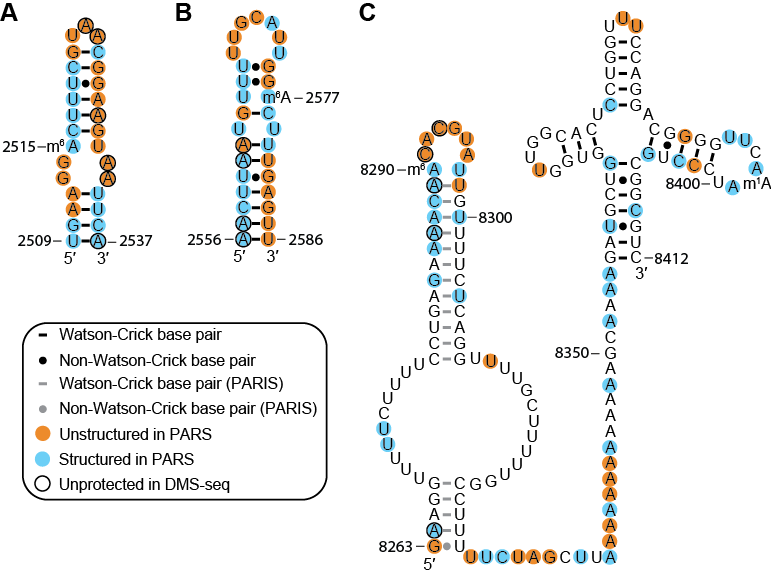


**Figure S1. PARS, DMS-seq, and PARIS data are consistent with previously determined structures of human MALAT1.** PARS, DMS-seq and PARIS data were mapped onto the schematics of established secondary structures for A) the hnRNPG-binding hairpin [12], B) the hnRNPC-binding hairpin [11], and C) the MALAT1 triple helix [17] and mascRNA [18]. All PARS, DMS-seq, and PARIS data are annotated as follows: Watson-Crick base pairing (line) and non-Watson-Crick base pairing (dots) are black unless detected by PARIS (gray lines and dots); nucleotide positions with orange and blue circles represent nucleotides that were unstructured and structured, respectively, in PARS; and a black outline denotes adenosine and cytidine residues considered unprotected in DMS-seq. m^6^A and m^1^A marks are labeled [55,59]. The paucity of PARS and DMS-seq data in nts 8263–8412 is likely because (i) both the triple helix and mascRNA structures are highly stable, making them insensitive to low amounts of nucleases [17,18], (ii) the triple helix is highly structured and bound by METTL16; therefore, few nucleotides would be modified by DMS [17,40], and (iii) mascRNA is a tRNA analog and tRNAs are refractory to RNA-seq, thereby requiring alternative detection methods [18,81]. After generating the composite model in Figure 2, the hnRNPG-binding hairpin is H63, the hnRNPC-binding hairpin is H64, the triple helix-containing hairpin is H190, and mascRNA is H191-H194.


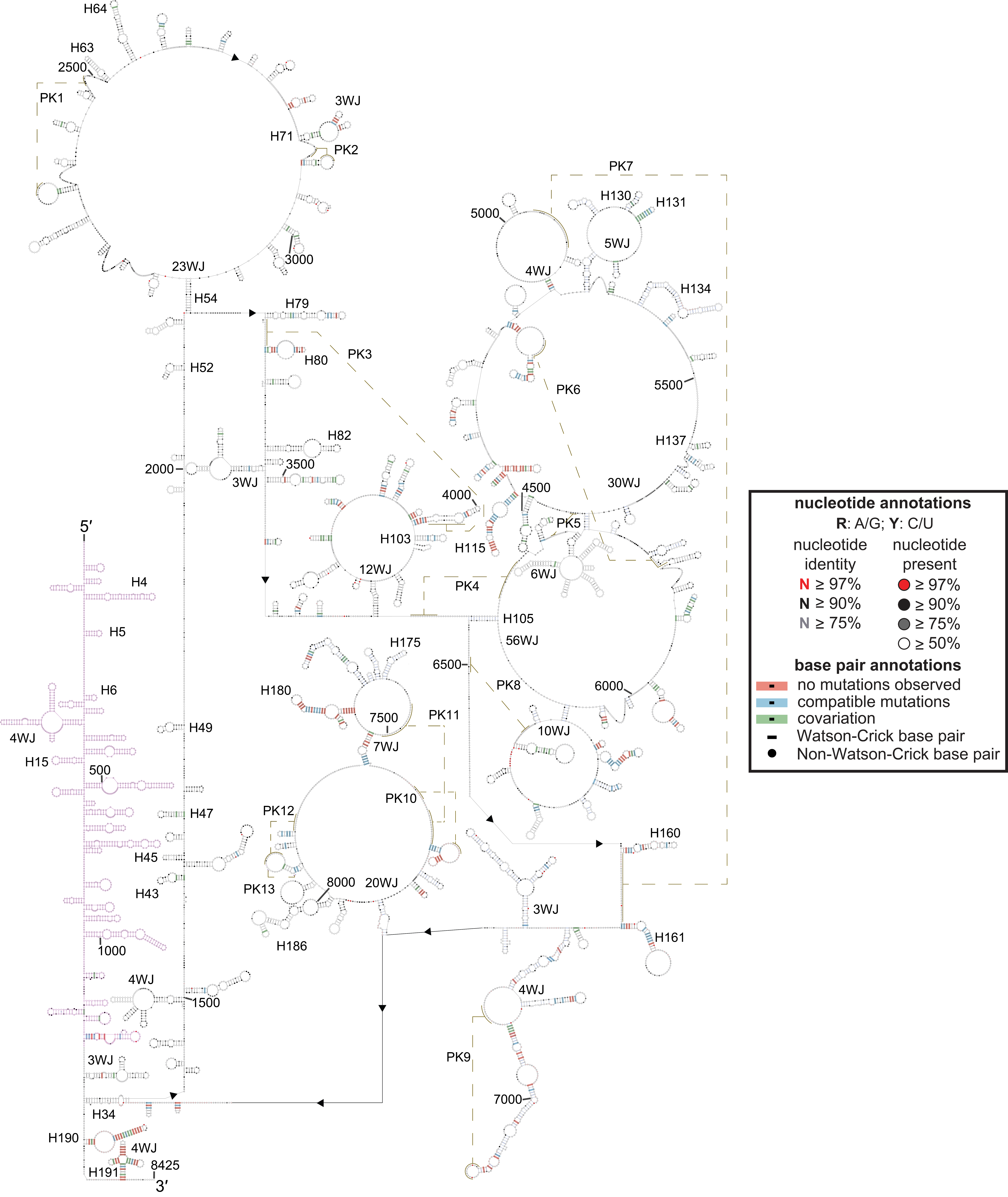


**Figure S2. Evolutionary sequence and structural conservation among 53 MALAT1 homologs.** Sequence conservation computed by R2R is denoted as follows: red nts are present in 97% or greater MALAT1 sequences, black nts are present in 90-96.9% MALAT1 sequences, gray nts are present in 75-89.9% MALAT1 sequences, red circles are nts present in 97% or greater MALAT1 sequences, black circles are nts present in 90-96.9% MALAT1 sequences, gray circles are nts present in 75-89.9% MALAT1 sequences, and white circles are nts present in 50-74.9% MALAT1 sequences. Structural conservation computed by R2R is denoted as follows: red boxes indicate 100% conserved base pair, blue boxes indicate compatible mutations for an indicated base pair, and green boxes indicate covariation for an indicated base pair. For clarity, only helices specifically mentioned in text are labeled; see Table S1 for a complete list of named structures and a list of conserved helices in the “Helices Coordinates” tab. Purple nucleotides indicate the secondary structure predicted using only MFE calculations. Yellow dashed lines indicate pseudoknot (PK) interactions.


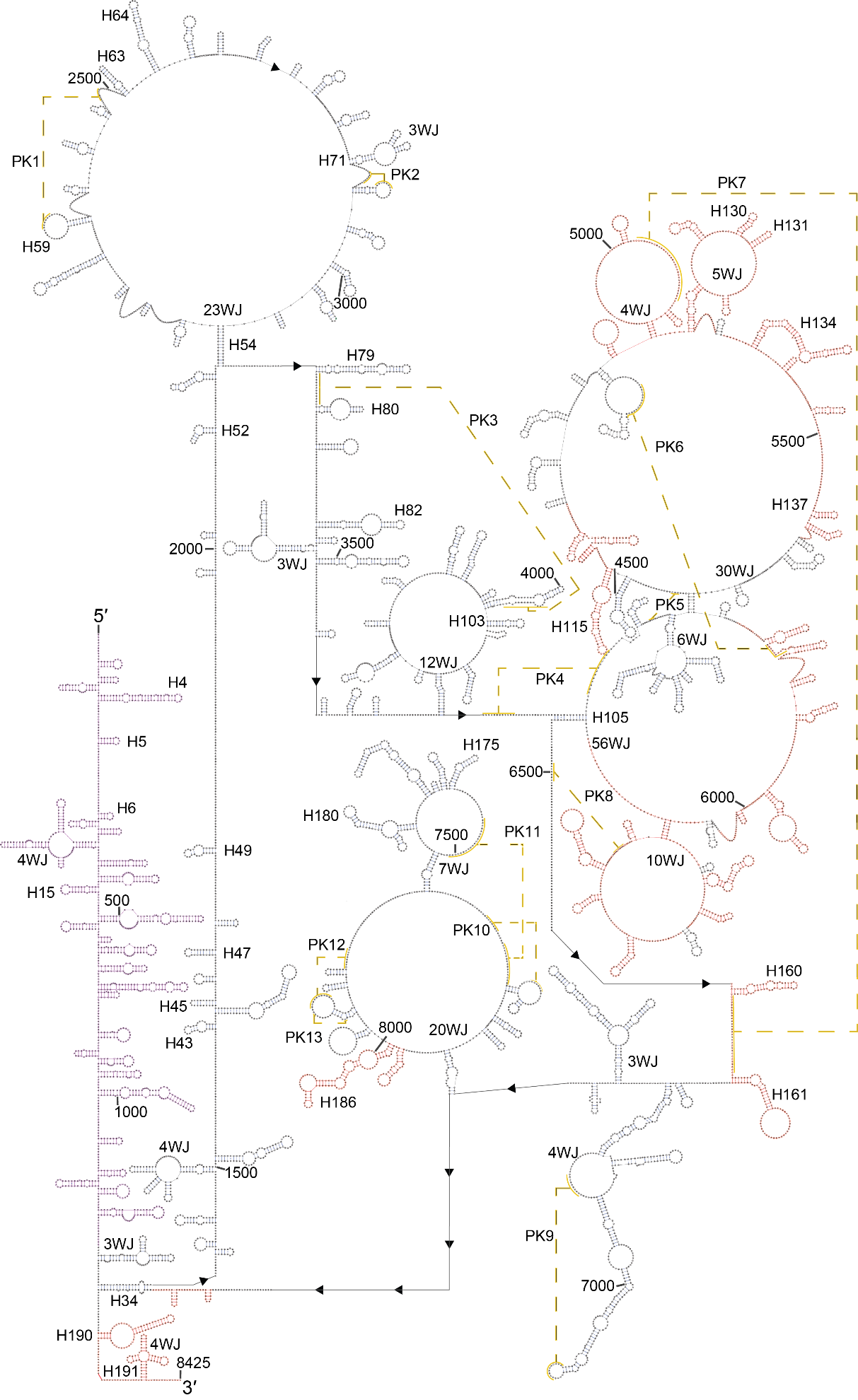


**Figure S3. Evolutionary core of MALAT1.** The secondary structures for the composite model is shown with red nucleotides denoting the evolutionary core of MALAT1 in vertebrates and purple nucleotides denoting the secondary structure predicted using only MFE calculations. For clarity, only helices specifically mentioned in the text are labeled; see Table S1 for a complete list of named structures. Solid lines with an arrowhead in the middle of the line denote 0 nt distance. Yellow dashed lines indicate RNA-RNA interactions in helices or pseudoknots.

**
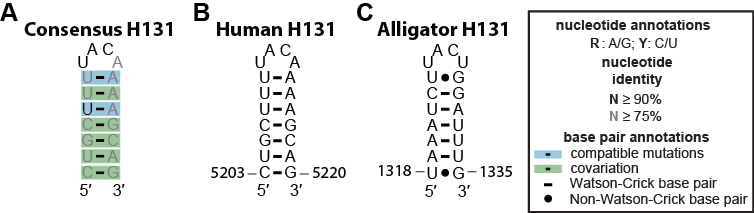
**

**Figure S4. Conservation analysis of H131 as determined by R2R.** Conservation, compatible mutations, and covariation are annotated as described in Figure S3 for A) the consensus H131 in MALAT1, B) human H131, and C) American alligator H131.

**Supplementary Table Legends**

**Table S1. Compiled data for construction and conservation analysis of the secondary structural model of human MALAT1.** In the “SeqMarkup” tab, previously published PARS (Lines 3-8) and DMS-seq (Line 12) data are displayed for MALAT1 at single-nucleotide resolution [21,22]. A ratio of RNase S1 to RNase V1 is provided in Line 11. Line 13 supplies the MFE model determined for nts 1–1280. Line 14 denotes the starting positions of previously published PARIS reads and line 15 denotes the nucleotides in PARIS reads that align as ssRNA (orange) or dsRNA (blue) [26,27]. The final nucleotide assignments as ssRNA (orange) or dsRNA (blue) are indicated for the composite model in line 16. Lines 17 and 18 annotate the agreement (i.e. 1 for agreement and 0 for no agreement) between PARS and DMS-seq data for ssRNA and dsRNA assignments. A direct comparison between PARS and Composite lines was determined for ssRNA (Line 19) and dsRNA (Line 20). Similarly, a direct comparison between DMS-seq and Composite lines was determined for unprotected (Line 21) and protected (Line 22) nts. The calculated values for these comparisons are in the “Statistics” tab. The “Helices Coordinates” tab contains a detailed list of numbered secondary structures as well as their genomic coordinates, nt numbers, and evolutionary conservation in at least 71% of primates, 82% of mammals, and 81% of vertebrates. The “Structured Loops” tab has a listing of the structured tetraloops and internal loops identified in our model. The “Rscape Significant BP” tab has a list of helices, number of base pairs, and nucleotide positions that correspond to statistically significant covarying base pairs identified by R-scape [28,33]. The “RNAStructureomeDB Agreement” tab replicated domain and z-score assignments from Andrews *et al.* [69] and lists the specific helices to which these domains correspond in our structural model of MALAT1.

**Table S2. Compiled data for binding partners and modifications of MALAT1.**

Tables of MALAT1-interacting partners and binding site information are included for proteins (“Proteins” tab), RNAs (“U1-rRNA-RPS6” tab), and miRNAs (“miRNA” tab and “miRNA Conservation” tab). Also included are tables of modified nucleotides (“Modified Nucleotides” tab), somatic mutations in cancer downloaded from the Genome Data Commons (“Somatic Mutations in Cancer” tab) [63], and SNPs (“SNPs” tab) [68].

Supplementary File Legends

**File S1.**

Infernal input containing FASTA-formatted file for all 53 annotated MALAT1 sequences and three negative controls used in the Infernal analysis.

**File S2.**

Infernal input containing a Stockholm alignment of MALAT1 homologs from *Homo sapiens* (human), *Pan paniscus* (bonobo), *Otolemur garnettii* (bushbaby), *Mus musculus* (mouse), and *Felis catus* (cat). This file served as an initial starting point for finding homologous MALAT1 structures in MALAT1 sequences from File S1.

**File S3.**

Infernal output containing a Stockholm-formatted file of observed homologous MALAT1 structures and sequences for nts 1–1280, with respect to the nucleotide numbering for the human MALAT1 sequence.

**File S4.**

Infernal output containing a Stockholm-formatted file of observed homologous MALAT1 structures and sequences for nts 1281–2000, with respect to the human MALAT1 sequence.

**File S5.**

Infernal output containing a Stockholm-formatted file of observed homologous MALAT1 structures and sequences for nts 2001–2999, with respect to the human MALAT1 sequence.

**File S6.**

Infernal output containing a Stockholm-formatted file of observed homologous MALAT1 structures and sequences for nts 3000–4000, with respect to the human MALAT1 sequence.

**File S7.**

Infernal output containing a Stockholm-formatted file of observed homologous MALAT1 structures and sequences for nts 4000–4950, with respect to the human MALAT1 sequence.

**File S8.**

Infernal output containing a Stockholm-formatted file of observed homologous MALAT1 structures and sequences for nts 4951–6000, with respect to the human MALAT1 sequence.

**File S9.**

Infernal output containing a Stockholm-formatted file of observed homologous MALAT1 structures and sequences for nts 6001–6550, with respect to the human MALAT1 sequence.

**File S10.**

Infernal output containing a Stockholm-formatted file of observed homologous MALAT1 structures and sequences for nts 6550–6800, with respect to the human MALAT1 sequence.

**File S11.**

Infernal output containing a Stockholm-formatted file of observed homologous MALAT1 structures and sequences for nts 6800–7360, with respect to the human MALAT1 sequence.

**File S12.**

Infernal output containing a Stockholm-formatted file of observed homologous MALAT1 structures and sequences for nts 7360–8000, with respect to the human MALAT1 sequence.

**File S13.**

Infernal output containing a Stockholm-formatted file of observed homologous MALAT1 structures and sequences for nts 8000–8250, with respect to the human MALAT1 sequence.

**File S14.**

Infernal output containing a Stockholm-formatted file of observed homologous MALAT1 structures and sequences for nts 8251–8425, with respect to the human MALAT1 sequence.
